# A meta‐analysis on shared and distinct neural correlates of the decision‐making underlying altruistic and retaliatory punishment

**DOI:** 10.1002/hbm.25635

**Published:** 2021-08-20

**Authors:** Sara Boccadoro, Lisa Wagels, Andrei A. Puiu, Mikhail Votinov, Carmen Weidler, Tanja Veselinovic, Zachary Demko, Adrian Raine, Irene Neuner

**Affiliations:** ^1^ Departments of Psychiatry, Psychotherapy, and Psychosomatics School of Medicine, RWTH Aachen University Aachen Germany; ^2^ JARA‐Institute Brain Structure Function Relationship (INM 10), Research Center Jülich Jülich Germany; ^3^ Department of Psychology University of Wisconsin‐Madison Madison Wisconsin; ^4^ Departments of Criminology, Psychiatry, and Psychology University of Pennsylvania Philadelphia Pennsylvania; ^5^ Institute of Neuroscience and Medicine (INM‐4) Forschungszentrum Jülich Jülich Germany; ^6^ JARA‐BRAIN ‐ Translational Medicine Aachen Germany

**Keywords:** aggression, altruism, magnetic resonance imaging, motivation, punishment, social norms

## Abstract

Individuals who violate social norms will most likely face social punishment sanctions. Those sanctions are based on different motivation aspects, depending on the context. Altruistic punishment occurs if punishment aims to re‐establish the social norms even at cost for the punisher. Retaliatory punishment is driven by anger or spite and aims to harm the other. While neuroimaging research highlighted the neural networks supporting decision‐making in both types of punishment in isolation, it remains unclear whether they rely on the same or distinct neural systems. We ran an activation likelihood estimation meta‐analysis on functional magnetic resonance imaging data on 24 altruistic and 19 retaliatory punishment studies to investigate the neural correlates of decision‐making underlying social punishment and whether altruistic and retaliatory punishments share similar brain networks. Social punishment reliably activated the bilateral insula, inferior frontal gyrus, midcingulate cortex (MCC), and superior and medial frontal gyri. This network largely overlapped with activation clusters found for altruistic punishment. However, retaliatory punishment revealed only one cluster in a posterior part of the MCC, which was not recruited in altruistic punishment. Our results support previous models on social punishment and highlight differential involvement of the MCC in altruistic and retaliatory punishments, reflecting the underlying different motivations.

## Graphical abstract

We ran an ALE meta‐analysis to investigate the neural correlates of decision‐making underlying social punishment and whether altruistic and retaliatory punishments share similar brain networks. Our results support previous models on social punishment and highlight the differential involvement of the midcingulate cortex in altruistic and retaliatory punishments, reflecting the underlying different motivations.

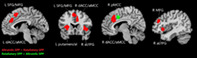

## 1 INTRODUCTION

Living in a dynamic social system requires a large degree of behavioral regulation based on social norms. When detecting social norms violations such as unfairness and inequity, individuals often react punitively toward social norm violators. The umbrella term for this behavior is social punishment (Zinchenko, 2019). In contrast to punishment by a neutral third person, punishment directly inflicted by the victim toward the perpetrator is labeled “second‐party punishment” (SPP, Fehr & Fischbacher, 2004). Importantly, SPP occurs without any benefit for the punisher other than re‐establishing the social norm, which depicts the primary motivation. If it entails a certain cost for the punisher, it is conceptualized as “altruistic punishment” (Du & Chang, 2015), wherein altruistic refers to the sacrifice of a personal gain to punish unfairness and to reinforce the social norm (Fehr & Gächter, 2002).

However, SPP may originate not only from a prosocial but also from a spiteful motivation (Brañas‐Garza, Espín, Exadaktylos, & Herrmann, 2014; Emmerling et al., 2016; Jensen, 2010; Yamagishi et al., 2017). Interestingly, the latter broadly matches retaliatory aggression. A retaliatory or reactive response is an aggressive response to provocation which is often driven by anger (Blair, 2004). Individuals tend to retaliate provocations and retaliation is even reciprocally increasing with higher provocations (Krämer, Jansma, Tempelmann, & Münte, 2007). We conceptualize this form of SPP originating from a spiteful motivation as “retaliatory SPP.” Importantly, provocation is a crucial component of SPP, as both altruistic and retaliatory forms of SPP are direct responses to provocation. Thus, when considering SPP and the cognitive processes underlying SPP, provocation must be taken into account, as any decision leading to SPP starts with experiencing and processing provocation. Consequently, it is hard to separate the pure act of punishment from provocation. As the present study focuses on decision‐making in SPP, and not on the punishment execution per se, this may also partly include decisions that are not representing a punishment (but its rejection). Whether they result in a punishment or not, the cognitive processes that follow a provocation deal with the decision about applying or not applying a punishment considering the gain and the costs of the potential punishment. Indeed, previous studies on altruistic SPP have shown a strong correlation between unfair offers (provocation) and rejected offers (punishment), meaning that unfair offers tend to be rejected (Camerer, 2003). The same strong correlation between provocation and punishment applies to retaliatory SPP. There is a well‐replicated positive association between provocation and retaliation, meaning that provocation reliably elicits retaliation (Konzok et al., 2020; Krämer et al., 2007; Weidler et al., 2019). Nevertheless, the strict distinction between scenarios in which a punishment would result in an altruistic decision and scenarios in which a punishment would be a retaliatory decision is artificial to a certain extent; in real‐world situations there can be different types and entities of cost for the punishment and more complex evaluations to be made that can result in an overlap of the two concepts. In this manuscript, we refer to the main focus of different laboratory scenarios (outlined further below) determining the motivation to punish an opponent.

Whether the motivational differences between altruistic and retaliatory SPP are reflected in distinct neurocognitive processes is currently unknown. Understanding the mechanisms involved in altruistic and retaliatory SPP is crucial for identifying the neural mechanisms that differentiate a more socially accepted and adaptive behavior (altruistic SPP) from retaliatory SPP, that is, especially in extreme forms, socially undesirable. Moreover, the latter is associated with violent behaviors and is often a transdiagnostic marker found across psychiatric disorders (Nelson & Trainor, 2007).

Different paradigms have been established to either, investigate altruistic SPP, or retaliatory SPP. To investigate altruistic SPP many studies used the Ultimatum Game (UG). The UG typically involves two players, one in the role of the proposer and one on the responder's role, who have to decide how to split a sum of money among each other (for a comprehensive account, see Güth, Schmittberger, & Schwarze, 1982). While the proposer freely decides how to split the money, the responder can only accept or reject the proposer's offer. Rejecting an offer means that neither the responder nor the proposer receives money. Since the responder is giving up on the money for the purpose of punishing norm violation (i.e., unfairness), rejecting an unfair offer represents altruistic SPP. Different aspects of altruistic SPP have been studied by modified versions of the UG including behavioral and neural measures (Civai, Crescentini, Rustichini, & Rumiati, 2012; Güroğlu, van den Bos, Rombouts, & Crone, 2010; Sanfey, Rilling, Aronson, Nystrom, & Cohen, 2003; Wei, Zhao, & Zheng, 2013).

Most established tasks to study retaliatory SPP are the Taylor Aggression Paradigm (TAP; Taylor, 1967) and the Points Subtraction Aggression Paradigm (PSAP; Cherek, Schnapp, Moeller, & Dougherty, 1996; Skibsted et al., 2017). In the TAP, participants compete for a reward with an ostensible opponent in a reaction time task. At the beginning of each trial, every player chooses a punishment level for the opponent that will become effective after the competition. The player who loses the competition will be punished as previously chosen by the other player. Different TAP versions employed various punishment modalities, including electric shocks (Taylor, 1967), money subtraction (Repple et al., 2017; Wagels et al., 2018, 2019; Weidler et al., 2019; Weidler, Habel, et al., 2019), noise blasts (Bushman, 1995; Krämer et al., 2007), or heat stimuli (Weidler, Habel, et al., 2019). Importantly, the decision about the punishment for the opponent does not relate to the earnings of the participant and neither does the participant have to sacrifice own earnings. In the PSAP, participants can choose to earn or steal points from the opponent and are repeatedly provoked by having their points stolen by the opponent. Unlike in the TAP, participants in the PSAP can escape the punishment by choosing to shield their points, instead of earning more or stealing the other's points. The number of stolen points is an indirect measure of retaliatory punishment but does neither come with a reward nor cost for the punishing participant.

Both the UG and the aggression paradigms involve a provocative behavior (e.g., receiving an unfair offer, punishment) by another person (proposer or opponent) and a response to such provocation (e.g., rejection of the unfair offer, retaliation, etc.). As such, both paradigms capture behaviors linked to SPP and the intent to signal disagreement with the opponent's behavior by punishing him/her. The costs for this punishment, however, are different in the UG compared with aggression tasks. Certainly, aggression tasks contain losses for the player in the form of provocation (as point losses in the PSAP, for instance). Administering the punishment to the opponent, however, brings no direct cost to the punisher. Thus, the costs and the retaliatory behavior are not directly related and there is no need to overcome self‐interest to carry out the punishment in aggression tasks successfully. In contrast, altruistic punishment in the UG requires overcoming self‐interest as for each rejected offer, the responder renounces a potential gain in order to punish the proposer. Consequently, we assume that the different underlying motivation differentiates altruistic SPP from aggression paradigms. To be specific, in the UG, participants have to pay for being able to punish. This corresponds to a decision for a superordinate goal, while the goal in aggression tasks is directly the punishment selection itself. From a conceptual perspective, this might be the key point that distinguishes decision‐making processes involved in altruistic from retaliatory SPP but it is unclear if this conceptual difference has a neural basis.

Neuronal networks play an important role in models that describe decision‐making in SPP. Previous research on altruistic SPP tentatively supports the norm violation model, according to which rejecting unfair offers corresponds to a rejection of norm violations (Civai et al., 2012; Corradi‐Dell'Acqua, Civai, Rumiati, & Fink, 2013; Gabay, Radua, Kempton, & Mehta, 2014). Specifically, Feng, Luo, and Krueger (2015) suggest that altruistic SPP is supported by two separable but interacting neuronal networks involved in fairness‐related norm enforcement (Buckholtz & Marois, 2012; Lieberman, 2007; Sanfey & Chang, 2008) as well as in decision‐making, as first proposed by Kahneman (2011): one that is reflexive and intuitive (System 1) and one that is deliberate (System 2). System 1 comprises the anterior insula and the ventromedial prefrontal cortex (vmPFC) and represents intuitive, automatic, and rapid responses to social norm violations. These responses collide with economic self‐interest and the ensuing conflict is encoded in the dorsal ACC (dACC), which then signals the need to resolve it to the second system (for a detailed review on dACC function, see Heilbronner & Hayden, 2016). The dACC is also referred to as anterior midcingulate cortex (aMCC), as defined by Vogt (2005) based on cytoarchitectonic information and structural and functional connectivity (see also Vogt, 2016). To prevent confusion, we will refer to this region as dACC/aMCC throughout this manuscript. Besides the dACC/aMCC, system 2 includes the ventrolateral prefrontal cortex (vlPFC), the left dorsolateral prefrontal cortex (dlPFC), the rostral ACC (rACC), and the right dlPFC and is involved in resolving the conflict. Selecting economic self‐interest and accepting unfair offers or over‐riding economic self‐interest and rejecting unfair offers are possible conflict resolution outcomes. Altruistic SPP can be observed if economic self‐interest is overridden and unfair offers are rejected, via activation of System 1, for evaluation of the norm violation, of the dACC/aMCC, to encode and signal the conflict with self‐interest and of the right dlPFC, to suppress self‐interest in order to punish.

This network model is partially supported by the meta‐analytical findings of Zinchenko (2019). The authors reported convergent bilateral activation in the anterior insula and left superior frontal gyrus (SFG, corresponding broadly to the dlPFC), areas related to salience and central‐executive networks. However, instead of dACC/aMCC activation, the authors found convergent activation of the right interior frontal gyrus (rIFG, corresponding to the ventrolateral PFC). This may highlight the involvement of emotional empathy required to understand others' intentions. Additionally, a meta‐analysis by Gabay et al. (2014) on the UG proposes a potential role of reward in punishing violations of social norms. Rejecting an unfair offer as a mean of punishment allows the participant to overcome the negative emotions elicited by inequality experience, which may be inherently satisfactory and rewarding.

Meta‐analytic findings on aggression paradigms suggest that activation of the left postcentral gyrus reflects action execution (Wong et al., 2019). Others allocate a network including the anterior insula/IFG and the ACC to aggressive actions (Puiu et al., 2020). These areas are part of the salience network involved in information decoding and preparation for action execution. In particular, the anterior insula/IFG detects salient information and forwards it to the ACC, which then integrates it in preparation for appropriate responses. Lastly, a recent meta‐analysis focusing on the role of the cerebellum in anger and threat processing and active aggression expression in healthy and clinical populations has shown involvement of the posterior cerebellum in anger and involvement of the anterior cerebellum in aggression (Klaus & Schutter, 2021). The posterior cerebellum is functionally associated with the somatomotor, the frontoparietal, and the default mode network (ACC, dlPFC, and vmPFC) and with the insula, the brainstem, and the angular gyrus. The anterior cerebellum is functionally coupled with somatomotor, somatosensory (spanning the postcentral gyrus), frontoparietal, limbic, and ventral attention network and with the posterior cingulate, that is part of the default mode network (Buckner, Krienen, Castellanos, Diaz, & Yeo, 2011; Klaus & Schutter, 2021). Given the extent of the functional connections of the cerebellum, its activation in anger processing and aggression expression may represent functional modulation of other areas involved in retaliatory SPP, such as the anterior insula/IFG, ACC, and postcentral gyrus reported in other meta‐analyses (Puiu et al., 2020; Wong et al., 2019).

To our knowledge, no meta‐analysis to date has compared the decision processes in altruistic and retaliatory SPP to elucidate their shared and different neural mechanisms. Neural patterns associated to these concepts may support our understanding of the similarity or differences of the respective motivational processes involved in decision‐making. Thus, meta‐analytically determining the functional divergence and convergence of both constructs is the primary interest and first aim of this review. Both types of punishment may differ in the neural substrates involved in overcoming self‐interest, which would be more pronounced in altruistic compared with retaliatory SPP. Specifically, we expect: (a) overlapping activation in the anterior insula/IFG and the dACC/aMCC, reflecting detection, decoding, and integration of the information related to the norm‐violation/provocative behavior; and (b) activation in the right dlPFC specific to altruistic SPP, reflecting overcoming self‐interest. Our second aim is to identify the networks generally involved in SPP, as these networks may support emotional processing and decision making in SPP. Our third aim is to update previous meta‐analytic findings on altruistic SPP assessed with the UG paradigm and on retaliatory SPP.

## 2 METHODS

### 2.1 Literature search and article selection

Studies were only included if, in the case of altruistic SPP, they involved SPP taking place in social interaction and the punishment occurred in response to unfair monetary splits. Retaliatory SPP studies were only included if they involved deliberate provocation, which resulted in retaliatory SPP. We excluded studies based on the following criteria: participants under 18 years old, irrelevant tasks, literature reviews, no reported fMRI data, only resting‐state fMRI data, single case studies, and no relevant contrasts/between‐group contrasts. Only studies reporting whole‐brain results for healthy adults were included. We selected our studies from two literature searches conducted in January 2021 on PubMed, one search for altruistic and one for retaliatory SPP. Study selection was conducted by two raters. In case of disagreement, a third rater was consulted for consensus.

**FIGURE 1:**
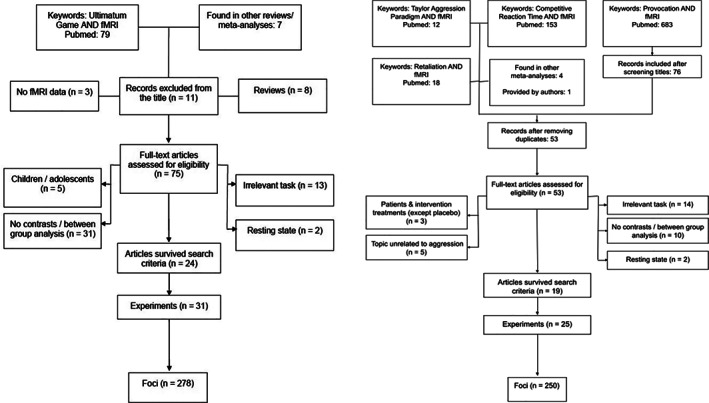
PRISMA flowcharts for the search and eligibility of the articles for the altruistic SPP analysis (on the left) and the retaliatory SPP analysis (on the right). The search was conducted in January 2021

For the altruistic SPP meta‐analysis, we conducted a standard search using the term “ultimatum game” in combination with “fMRI,” which yielded *n* = 79 studies [entire search string: (ultimatum game) AND (fMRI)]. We found seven additional studies in review articles. After abstract screening and checking for duplicates 11 articles were excluded resulting in 75 articles for full‐text review. After scanning for exclusion criteria, 24 studies with 30 experiments remained (22 unfair > fair and 8 reject > accept) including 692 subjects and 275 foci. We additionally contacted authors of the included manuscript to request data on the reject > accept contrast when not available in the published manuscripts. One author could provide the requested data, resulting in a total of 24 studies with 31 experiments (22 unfair > fair and 9 reject > accept) including 692 subjects and 278 foci (Figure 1). All studies included for altruistic punishment used either the ultimatum game or modified versions thereof. More details on the selection process, including number of studies excluded and reasons for the exclusion are available in the PRISMA flowchart (Figure 1).

For the retaliatory SPP meta‐analysis, we conducted a standard search using the terms “Taylor aggression paradigm,” “competitive reaction time,” “retaliation,” and “provocation” in combination with “fMRI” [entire search string: (Taylor aggression paradigm) AND (fMRI); (competitive reaction time) AND (fMRI); (retaliation) AND (fMRI); (provocation) AND (fMRI)]. These searches yielded a total of *n* = 683 studies. We found four additional studies in review articles. One additional study, not published yet, was kindly provided by authors of our team. The high number of studies was first screened based on article titles and abstract that clearly pointed to unrelated topics (e.g., provocation of disease symptoms instead of social provocation). After removing duplicates and not topic related articles, 83 studies were screened in a full‐text review. This yielded 19 studies with 25 experiments (9 feedback phase, 12 decision phase, and 4 in which the two phases were not separated) on 659 subjects, and 250 foci that entered analysis (Figure 1). Most studies included for retaliatory SPP used the Taylor aggression paradigm (79%) with the remaining four using the social network aggression task, the fight‐or‐escape task, the Pain Stimulation Task and the PSAP. More details on the selection process, including number of studies excluded and reasons for the exclusion are available in the PRISMA flowchart (Figure 1).

Following Müller et al. (2018) recommendations, only experiments reporting results in a standard stereotactic reference space (MNI, Talairach) were included in our study and converted into the same space. All studies we planned to include reported coordinates in standard stereotactic reference space, so no study was excluded for this reason. Talairach coordinates were converted into MNI coordinates using the icbm2tal transformation developed by Lancaster et al. (2007) and then used for the meta‐analysis. In total, data from 1,351 subjects (692 for altruistic punishment and 659 for retaliatory punishment) were included in the analyses. The sample in altruistic SPP studies consisted of approximately 40% males and 60% females and had a mean age of 28 years. The sample in retaliatory SPP studies consisted of approximately 50% males and 50% females and had a mean age of 23 years. An overview of the studies included in this meta‐analysis is outlined in Table 1.

**TABLE 1:** Summarized information of studies and experiments included in the meta‐analysis

Study	Age group (M age ± *SD*)	*N*	Sex	Handedness	Task	Contrast	Final contrast	# foci
*Altruistic SPP*								
Baumgartner, Knoch, Hotz, Eisenegger, and Fehr (2011)	21.6 ± 2.2	32	All M	All R	UG	Unfair > fair	Unfair > fair	17
Cheng et al. (2015)	21.6 ± 3.1	32	23 F, 9 M	All R	UG and IG	∩ unfair–fair (UG) and unfair–fair (IG)	Unfair > fair	7
						∩ reject unfair–accept unfair (UG) and reject unfair–accept unfair (IG)	Reject > accept	10
Civai et al. (2012)	n/r	19	12 F, 7 M	n/r	Mod. UG/DG	Unequal > equal	Unfair > fair	5
						Reject > accept	Reject > accept	2
Corradi‐Dell'Acqua et al. (2013)	23.5 (18–35)	23	9 F, 12 M	n/r	UG	Rejected > accepted	Reject > accept	2
Cortes et al. (2018)	40.5	27	8 F, 19 M	All R	UG	Unfair > fair	Unfair > fair	2
Fatfouta, Meshi, Merkl, and Heekeren (2018)	24.35 ± 3.80	23	8 F, 15 M	n/r	UG	Unfair UNKNOWN > fair UNKNOWN	Unfair > fair	8
Gospic et al. (2011)	23.7 ± 4.2	17	5 M, 12 F	All R	UG	Unfair > fair	Unfair > fair	4
Gradin et al. (2015)	25.48 ± 5.52	23	8 M, 17 F (pre‐exc.)	n/r	UG	Increasing inequality (decreasing fairness)	Unfair > fair	10
Guo et al. (2013)	22.44 ± 3.49	21	17 F, 10 M (pre‐exc.)	All R	UG	Unfair > fair	Unfair > fair	13
						Reject > accept	Reject > accept	18
Guo et al. (2014)	21.06 ± 2.10	18	13 F, 5 M	All R	UG	Unfair > fair	Unfair > fair	10
Güroğlu et al. (2010)	20.4 ± 1.7	23	13 F, 10 M	All R	Mod. UG	Rejection > acceptance	Reject > accept	7
Halko, Hlushchuk, Hari, and Schürmann (2009)	29	23	8 F, 15 M	21 R, 2 L	UG	Small offers > large offers (no‐competition)	Unfair > fair	12
Harlé and Sanfey (2012)	22.4 64.1	18 20	10 F, 8 M 13 F, 7 M	n/r	UG	Unfair > fair	Unfair > fair	12
Hu et al. (2016)	21.22 ± 1.73	23	13 F, 10 M	All R	UG	Unfair > fair	Unfair > fair	3
Kirk, Downar, and Montague (2011)	36.8 ± 10.1	40	21 F, 19 M	n/r	UG	Unfair > fair	Unfair > fair	11
Roalf (2010)	27.92 ± 2.66 71.71 ± 3.29	13 young 14 old	7 F, 6 M 7 F, 7 M	n/r	UG	Unfair > fair	Unfair > fair	8
Sanfey et al. (2003)	21.8 ± 7.8	19	11 F, 8 M	n/r	UG	Unfair > fair	Unfair > fair	17
Servaas et al. (2015)	20.8 ± 2.0	120	All F	All R	UG	Unfair > fair	Unfair > fair	32
						Unfair rejected > unfair accepted	Reject > accept	6
Wei et al. (2018)	22.46 ± 2.62 (pre‐exc.)	25	15 F, 10 M	All R	Mod. UG	Unfair > fair	Unfair > fair	6
						Unfair rejected > unfair accepted	Reject > accept	5
White, Brislin, Sinclair, and Blair (2014)	28.1 ± 8.1	21	9 F, 12 M	n/r	Mod. UG	Regions increasing in activity as offer unfairness increased^a^	Unfair > fair	7
						Regions increasing in activity as punishment increased^a^	Reject > accept	12
Wu, Zang, Yuan, and Tian (2015)	22.31 ± 2.35 (pre‐exc.)	27	24 F, 8 M (pre‐exc.)	All R	UG followed by DG	Unfair > fair	Unfair > fair	1
Zheng et al. (2015)	21.44 ± 3.38	25	18 F, 7 M	All R	UG	Unequal > equal	Unfair > fair	15
Zheng et al. (2017)	22.8 ± 1.4 (pre‐exc.)	18	12 F, 9 M (pre‐exc.)	n/r	UG	Unfair > fair	Unfair > fair	9
Zhou, Wang, Rao, Yang, and Li (2014)	25.07 ± 3.35	28	15 F, 13 M	n/r	UG	Unfair > fair	Unfair > fair	4
						Reject > accept	Reject > accept	3
*Retaliatory SPP*								
Achterberg, van Duijvenvoorde, Bakermans‐Kranenburg, and Crone (2016)	22.63 ± 2.62	30	15 F, 15 M	All R	SNAT	Negative > neutral feedback	Feedback phase	12
Beyer, Münte, Erdmann, and Krämer (2014)	23.2 ± 2.7 (pre‐exc.)	30	All F	27 R, 3 L (pre‐exc.)	TAP	High > low provocation	Decision phase	2
Brunnlieb, Münte, Krämer, Tempelmann, and Heldmann (2013)	25 ± 4.0 (pre‐exc.)	15	All M	All R	TAP	Active > passive trials	Decision phase	29
Buades‐Rotger, Beyer, and Krämer (2017)	22 ± 4	36	All F	n/r	FOE	Fight > avoid	Decision phase	11
						High > low provocation		23
Chester and DeWall (2016)	18.7 ± 0.93	69	47 F, 22 M	n/r	TAP	Retal. > non‐retal. aggression	Feedback and decision phase	18
Chester and DeWall (2018)	23.04 ± 2.46	24	13 F, 11 M	All R	TAP	Retal. > non‐retal. aggression	Feedback and decision phase	2
Chester and DeWall (2019)	18.61 ± 0.84	61	44 F, 17 M	All R	TAP	High > low provocation	Feedback phase	8
Chester et al. (2019)	18.98 ± 1.07	61	37 F, 24 M	All R	TAP	Retal. > non‐retal. aggression	Feedback and decision phase	4
Chester, Lynam, Milich, and DeWall (2018)	20.28 ± 2.77	60	38 F, 22 M	All R	TAP	Retal. > non‐retal. aggression (positive association)	Feedback and decision phase	2
Dambacher et al. (2015)	22.33 ± 2.35	15	All M	n/r	TAP	Provocation > no provocation	Decision phase	10
Emmerling et al. (2016)	22.33 ± 2.35	15	All M	n/r	TAP	Provocation > no provocation	Decision phase	5
Korotkov et al. (n.d.)	24.5 ± 3.6	39	26 F, 13 M	n/r	TAP	Scale: High > low provocation	Decision phase	7
						Feedback: High > low provocation	Feedback phase	6
Krämer et al. (2007)	22.9 ± 2.2 (pre‐exc.)	15	11 F, 9 M (pre‐exc.)	All R	TAP	High > low provocation	Decision phase	20
Lotze, Veit, Anders, and Birbaumer (2007)	28.6 ± 6.5 (pre‐exc.)	14	All M	n/r	TAP	Receiving aversive stimuli	Feedback phase	16
						Retaliation	Decision phase	3
Repple et al. (2017)	23.6 ± 3.2 (pre‐exc.)	29	All M	All R	TAP	Aggression after high provocation > aggression after low provocation	Decision phase	4
						High > low provocation	Feedback phase	2
Skibsted et al. (2017)	24.6 ± 2.9	19	11 F, 8 M	n/r	PSAP	Provocation event > monetary reward	Feedback phase	2
						Aggressive response > monetary reward	Decision phase	3
						Stealing reward > monetary reward		3
Wagels et al. (2019)	23.96	42	All M	All R	TAP	Feedback: High > low provocation	Feedback phase	3
						Decision: High > low provocation	Decision phase	1
Weidler, Wagels, et al. (2019)	24.86 ± 3.92 (pre‐exc.)	52	All M	All R	TAP	Decision phase: Parametric modulation “decision provocation previous”	Decision phase	19
						Provocation phase: Parametric modulation “provocation intensity”	Feedback phase	22
Yu, Li, and Zhou (2015)	22	33	16 F, 17 M	All R	PST	Harm–no‐harm > No‐harm–No‐harm	Feedback phase	6
						Intention effect		6

Abbreviations: ∩, conjunction analysis; DG, dictator game; F, females; FOE, fight‐or‐escape task; IG, impunity game; L, left; M age, mean age; M, males; Mod., modified; n/r, not reported; N, number of subjects; pre‐exc., pre‐exclusion; PSAP, points subtraction aggression paradigm; PST, pain stimulation task; R, right; SD, standard deviation; SNAT, social network aggression task; SPP, second‐party punishment; TAP, Taylor aggression paradigm; UG, ultimatum game.

^a^ Indicates parametric modulation.

### 2.2 ALE meta‐analysis

We used the GingerALE software, V3.0.2 (http://brainmap.org/software.html#GingerALE, RRID:SCR_014921) to conduct coordinate‐based meta‐analyses using the revised activation‐likelihood estimation (ALE) algorithm (Turkeltaub et al., 2012) of Eickhoff et al. (2009). This method uses foci from different studies to calculate a probabilistic map of activations that is compared against an expected null spatial distribution at the voxel‐level. The ALE algorithm first creates 3D images for each group of pooled foci computed using the input foci, a mask defining the outer limit of MNI space, and a Gaussian distribution with a Full‐Width Half‐Maximum (FWHM) calculated from the experiments' sample size. The ensuing images are modeled activation (MA) maps containing voxel‐wise ALE scores. These maps are then combined to create the ALE image that represents the functional overlap between sets of foci at each voxel. This overlap has to occur above chance level in order to represent true between‐experiment convergence. Last, the convergence of foci is tested by comparing the ALE scores against the null‐hypothesis of random spatial brain activations (Eickhoff et al., 2016; Turkeltaub et al., 2012).

We ran five separate meta‐analyses: (a) one global analysis for SPP across all subjects and experiments combining altruistic and retaliatory SPP, (b) one global analysis on altruistic SPP alone combining reject > accept and unfair > fair contrasts, (c) one global analysis for retaliatory SPP alone combining retaliation > no retaliation and high provocation > low provocation contrasts, (d) one conjunction analysis to determine the functional convergence between altruistic and retaliatory SPP, and (e) one contrast analysis to determine differences in the convergence between altruistic and retaliatory SPP. Significance has been assessed using a cluster‐level family‐wise error (cFWE) correction set at *p* < .05 with an uncorrected cluster‐forming threshold of *p* < .001 and 5,000 permutations. The cluster extent threshold was determined automatically by the software.

Due to the strong positive relationship between provocation and punishment (Camerer, 2003; Konzok et al., 2020; Krämer et al., 2007; Weidler, Habel, et al., 2019), we combined the unfair > fair and the reject > accept experiments in the altruistic SPP analysis and the feedback phase and the decision phase experiments, as well as experiments not separating between the two phases, in the retaliatory SPP analysis. Consequently, while the analyses on altruistic SPP and retaliatory SPP do not capture brain activation related exclusively to the punishment execution, they are able to determine which brain regions are activated throughout the whole decision‐making process that might lead to punishment. With the same rationale, we ran the global analysis on SPP combining all contrasts. In particular, the altruistic SPP experiments (unfair > fair and reject > accept) were combined in one single experiment per study (altruistic SPP experiment) resulting in 24 altruistic SPP experiments. The same applies to retaliatory SPP (feedback phase, decision phase, feedback, and decision phase combined) resulting in 19 experiments. Therefore, the global analysis on SPP included 43 experiments.

We also attempted to separate between the feedback and the decision in both types of SPP in explorative analyses. As such, we investigated convergence in activation related to (6) the unfair > fair contrast and (7) the reject> accept contrast, which correspond, respectively, to the feedback phase and the decision phase in altruistic SPP. Similarly, we analyzed the (8) the feedback phase and (9) the decision phase contrasts in retaliatory SPP. Lastly, because the TAP is the most used paradigm to investigate retaliatory SPP, we ran a subanalysis on (10) retaliatory SPP including only the subset of studies using the TAP. Since the number of experiments included in these subanalyses (with the except of the unfair > fair analysis and the TAP analysis) does not meet the minimum requirements for performing an ALE meta‐analysis (see Müller et al., 2018), these results should be interpreted with caution. The results for the subanalyses are available in Appendix S1.

## 3 RESULTS

### 3.1 Second‐party punishment

**FIGURE 2:**
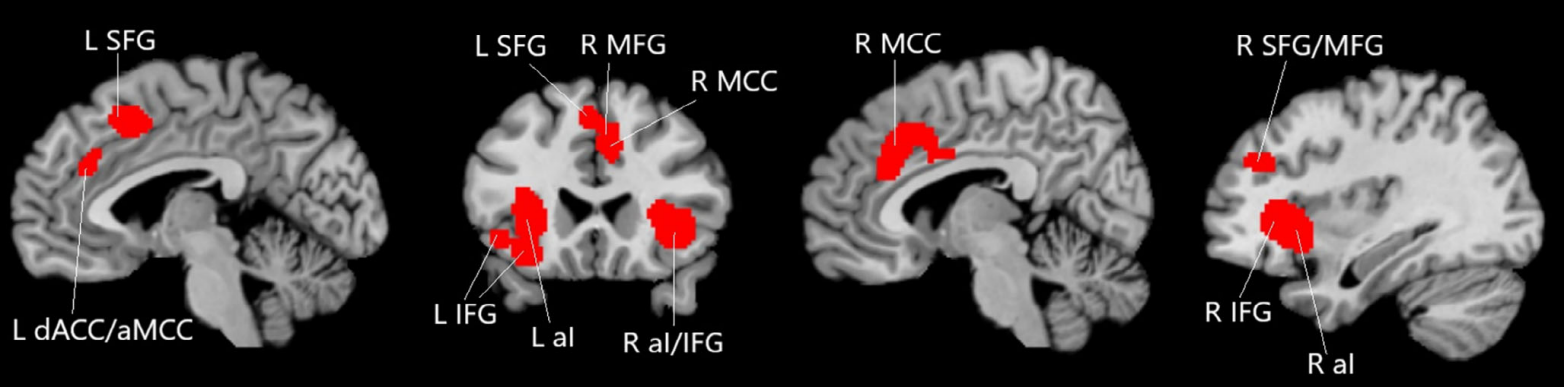
Results for the meta‐analysis on SPP. aI, anterior insula; aMCC, anterior midcingulate cortex; dACC, dorsal anterior cingulate cortex; IFG, inferior frontal gyrus; L, left; MCC, midcingulate cortex; MFG, medial frontal gyrus; R, right; SFG, superior frontal gyrus

The global meta‐analysis for SPP across all 1,351 subjects and 43 experiments revealed four clusters of consistent activity comprising (a) the left anterior insula/IFG, (b) the bilateral dACC/MCC and left SFG, (c) the right anterior insula/IFG, and (d) the right MFG/SFG (Table 2; Figure 2).

**TABLE 2:** Results for the meta‐analysis on SPP

*n*	Brain regions	BA	MNI coordinates (mm)				
			*x*	*y*	*z*	ALE score	*k*
#1	L aI	13	−32	22	4	0.0530	6,808
	L frontal lobe	47	−32	20	−16	0.0387	
	L aI	13	−36	16	−6	0.0252	
	L IFG	47	−44	20	−10	0.0248	
	L IFG	47	−30	32	−18	0.0201	
#2	L SFG	6	−4	18	48	0.0404	5,896
	R dACC/aMCC	32	6	26	34	0.0395	
	R dACC/aMCC	32	8	30	24	0.0287	
	L dACC/aMCC	32	−6	36	24	0.0225	
	R pMCC	32	4	14	38	0.0219	
	R pMCC	24	6	2	30	0.0184	
	R pMCC	24	6	10	30	0.0164	
#3	R aI/IFG		32	26	2	0.0543	5,776
	R aI/IFG		38	22	−4	0.0482	
#4	R MFG	9	40	34	26	0.0263	1,008
	R SFG		32	40	28	0.0199	

Abbreviations: aI, anterior insula; aMCC, anterior midcingulate cortex; BA, Brodmann area; dACC, dorsal anterior cingulate cortex; IFG, inferior frontal gyrus; *k*, cluster size; L, left; MFG, medial frontal gyrus; *n*, cluster number; pMCC, posterior midcingulate cortex; R, right; SFG, superior frontal gyrus.

### 3.2 Altruistic SPP

The meta‐analysis for altruistic SPP (692 subjects, 30 experiments) revealed four clusters of consistent activity comprising (a) the bilateral dACC/aMCC and left SFG, (b) the left anterior insula/IFG, (c) the right anterior insula/IFG, and (d) the right MFG (Table 3).

**TABLE 3:** Results for the meta‐analysis on altruistic SPP

*n*	Brain regions	BA	MNI coordinates (mm)			ALE value	*k*
			*x*	*y*	*z*		
#1	L SFG	6	−4	18	48	0.0396	6,784
	R dACC/aMCC	32	6	26	34	0.0396	
	R dACC/aMCC	32	8	30	24	0.0265	
	L dACC/aMCC	32	−6	36	24	0.0222	
	L dACC/aMCC	32	−6	30	32	0.0205	
#2	L aI	13	−30	22	4	0.0519	5,192
	L aI	13	−36	16	−6	0.0235	
	L IFG	47	−32	22	−16	0.0196	
	L IFG	47	−46	18	−12	0.0153	
#3	R aI/IFG	13	34	26	0	0.0440	5,024
	R aI/IFG		38	22	−4	0.0437	
#4	R MFG	9	40	34	26	0.0251	960

Abbreviations: aI, anterior insula; aMCC, anterior midcingulate cortex; BA, Brodmann area; dACC, dorsal anterior cingulate cortex; IFG, inferior frontal gyrus; *k*, cluster size; L, left; MFG, medial frontal gyrus; *n*, cluster number; R, right; SFG, superior frontal gyrus.

### 3.3 Retaliatory SPP

The meta‐analysis for retaliatory SPP (659 subjects, 23 experiments) revealed one cluster of consistent activity strongly right lateralized in the posterior MCC (pMCC; Table 4).

**TABLE 4:** Results for the meta‐analysis on retaliatory SPP

*n*	Brain regions	BA	MNI coordinates (mm)			ALE score	*k*
			*x*	*y*	*z*		
#1	R pMCC	32	4	14	38	0.0195	960
	R pMCC	24	8	8	33	0.0184	
	R pMCC	24	8	8	42	0.0169	

Abbreviations: BA, Brodmann area; *k*, cluster size; *n*, cluster number; pMCC, posterior midcingulate cortex; R, right.

### 3.4 Conjunction analysis

The conjunction analysis revealed no cluster of convergent activity between altruistic and retaliatory SPP.

### 3.5 Contrast analysis

**FIGURE 3:**
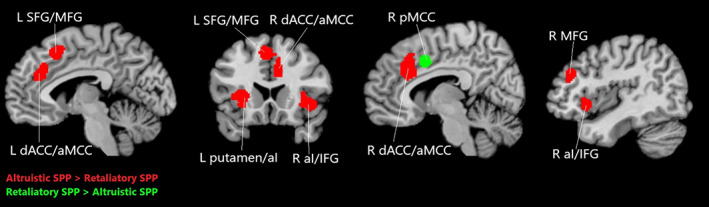
Results for the contrast analysis. In red the results for altruistic SPP > retaliatory SPP, in green the results for retaliatory SPP > altruistic SPP. aI, anterior insula; aMCC, anterior midcingulate cortex; dACC, dorsal anterior cingulate cortex; IFG, inferior frontal gyrus; L, left; MFG, medial frontal gyrus; pMCC, posterior midcingulate cortex; R, right; SFG, superior frontal gyrus

Altruistic SPP in contrast to retaliatory SPP revealed more substantial convergence of activation in four clusters, including (a) the bilateral dACC/aMCC and SFG/MFG, (b) the right anterior insula/IFG, (c) the putamen and left anterior insula, and (d) the right MFG (Table 5; Figure 3).

**TABLE 5:** Results for the contrast analysis between altruistic SPP and retaliatory SPP

*n*	Brain regions	BA	MNI coordinates (mm)			
			*x*	*y*	*z*	*k*
#1	R dACC/aMCC	32	8.8	25.7	30.8	5,200
	L SFG	8	−4.7	21.3	48	
	L SFG	6	−6	16	52	
	R SFG	8	2	24	48	
	L dACC/aMCC	32	−6	32.4	28.4	
	L MFG	9	−8	33	29	
	L dACC/aMCC	32	−8	38	26	
#2	R aI/IFG	13	38	22	0	2040
#3	L putamen/aI		−24	18	4	1,608
	L putamen/aI		−26	16	0	
#4	R MFG	9	42	30	28	600
	R MFG	9	44	34	30	
	R MFG	9	44	37	23	
	R MFG	9	44	32	22	
*Retaliatory SPP > altruistic SPP*						
#1	R pMCC	24	8	4	42	640
	R pMCC	24	6.7	7.3	38	

Abbreviations: aI, anterior insula; aMCC, anterior midcingulate cortex; BA, Brodmann area; dACC, dorsal anterior cingulate cortex; IFG, inferior frontal gyrus; *k*, cluster size; L, left; MFG, medial frontal gyrus; *n*, cluster number; pMCC, posterior midcingulate cortex; R, right; SFG, superior frontal gyrus.

In contrast to altruistic SPP, retaliatory SPP revealed only one cluster corresponding to the right pMCC (Table 5; Figure 3).

## 4 DISCUSSION

This study used ALE meta‐analyses to identify the shared and distinct neural correlates of decision‐making in altruistic and retaliatory SPP. Further, we aimed to shed light on the network supporting SPP decision‐making and update previous findings on altruistic and retaliatory SPP. The conjunction analysis did not show significant convergent activation related to the two concepts. The global analysis, however, showed that SPP reliably recruited activation in the bilateral insula/IFG, the bilateral MCC/dACC, and the bilateral MFG/SFG. This network largely corresponds to regions activated by altruistic SPP but extends to the right pMCC as activated in retaliatory SPP. This is comparable with what reported by Puiu et al. (2020), who included retaliatory SPP tasks together with altruistic SPP and third‐party punishment tasks.

Contrary to our hypothesis, we did not find overlapping activation in the anterior insula/IFG and dACC/aMCC for altruistic and retaliatory SPP. Instead, the contrast analysis revealed that altruistic, compared with retaliatory SPP, showed more convergent activation in the cortical and subcortical regions involved in SPP. Retaliatory SPP tasks, on the other hand, revealed only one cluster located in a posterior portion of the right MCC showing more convergent activation compared with altruistic SPP tasks. Our findings support the assumption of specific and divergent neural networks which may underlie different motivations in SPP. Decision‐making in altruistic SPP, driven by the large conflict between the social norm of fairness and self‐interest, might require more cognitive resources to resolve the conflict between the two different motives and decide whether to execute the punishment (Sanfey et al., 2003). In particular, the activation of the right dlPFC in altruistic and not in retaliatory SPP supports our hypothesis that this area activates specifically during altruistic SPP. We speculate that this may indicate overcoming self‐interest. In contrast, retaliatory SPP originates from a spiteful motivation, which is less cognitively demanding as there is less conflict to resolve, related to the punishment level to administer, but not concerning self‐interest.

Our findings on altruistic SPP revealed a network that overlaps with previous meta‐analytic findings (Feng et al., 2015; Gabay et al., 2014; Zinchenko, 2019), in particular with regions mentioned in the two‐system model for punishing norm violations (Feng et al., 2015). We found convergent activation in the anterior insula/IFG, which according to the two‐systems model, belongs to System 1 and is assumed to represent intuitive responses. Furthermore, we also found dACC/aMCC and MFG/SFG (bilateral dlPFC) activation, belonging to System 2, which is thought to manage conflict. The separate subanalyses on the feedback (unfair > fair) and the decision phase (reject > accept) showed that the whole network identified in the main analysis is active at the feedback phase level before the decision. This likely shows that the decision‐making process underlying altruistic SPP starts already with the provocation. However, this speculative assumption has yet to be investigated. The decision phase showed activation only in the left aI/IFG/putamen. Yet, the decision phase analysis was based on a limited number of experiments (nine), which might explain the lack of convergent activation in the other regions of the altruistic SPP network.

Crucially, dACC/aMCC involvement was reported in previous meta‐analyses (Feng et al., 2015; Gabay et al., 2014), but not by Zinchenko (2019). This might be due to the heterogeneity of the studies that were included in the meta‐analysis and did not always involve resolving a cognitive conflict (UG, third‐party punishment game, criminal scenarios evaluation, and social rejection tasks). Our findings, however, support the involvement of the dACC/aMCC specifically in altruistic SPP. The dACC/aMCC is involved in regulating cognitive control by providing continuous updated account of cognitive demand based on changes in the situational complexity (Sheth et al., 2012). This function allows the dACC/aMCC to guide reward‐based decision making, to predict task difficulty, and to monitor conflict. While not excluding other functions of the dACC/aMCC, a cognitive demanding conflict underlying the motivation in the altruistic SPP decision‐making would be in line with this convergent activation pattern in this region. We speculate in line with the two neuronal network model, that dACC/aMCC recruitment may be needed to monitor the conflict between unfair offers and self‐interest and to guide decision‐making in punishing unfairness.

In addition, we found recruitment of the putamen specifically in altruistic SPP compared with retaliatory SPP, which is in line with findings by Gabay et al. (2014). Given that the putamen is often involved in reward processing (Arsalidou, Vijayarajah, & Sharaev, 2020), the authors hypothesized that activation in this region might show a potential role of reward in altruistic SPP. As inherently rewarding, the possibility to punish experienced inequality likely supports this decision more than self‐interest. In other words, the motivation to re‐establish the social norm by punishing unfairness should be rewarding enough to overcome self‐interest. For a successful conflict resolution, the decision process requires cognitive resources to evaluate the best between the two possible outcomes. However, the potential role of reward in altruistic SPP needs to be investigated in future studies involving rewards‐specific tasks manipulations.

Retaliatory SPP yielded convergent activation in a more posterior portion of the MCC compared with that found in altruistic SPP. The pMCC is involved in multisensory orientation of the body in space in response to sensory stimuli such as pain, and more generally in the processing of motor and pain information (Vogt, 2016; Yu et al., 2011). A Neurosynth search (http://neurosynth.org, RRID:SCR_006798) of the peak coordinate for retaliatory SPP indicates that this area associated with motor control and pain processing. While the meta‐analysis cannot disentangle specific functions during the punishment decision process, it is conceivable that the pMCC might be recruited in retaliatory SPP tasks to process the provocation stimuli and prepare a fast motor response without much rumination or cognitive demanding evaluation of the social context. In absence of a direct cost for the participant, the decision to punish would not require solving a conflict between the provocation and self‐interest and the punishment can be administered more directly, after solving only the conflict related to choosing a high or low punishment. If this role of the pMCC in retaliatory SPP were true, its activation might be mostly related to the feedback phase, where participants evaluate the provocation and prepare a response. Indeed, the subanalysis on the feedback phase indicates an activation of the pMCC in this phase. However, these results are based on a low number of experiments (nine), which is not considered reliable for drawing any robust conclusion. Future studies should aim at separating the feedback phase and the decision phase to pinpoint which regions support the response preparation and those recruited during the actual punishment selection.

The differential recruitment of the aMCC/dACC and pMCC in altruistic and retaliatory SPP might thus reflect differential motivation involved in the decision‐making processes underlying the two types of SPP, with a cognitive demanding conflict resolution motivation in altruistic SPP and spiteful motivation in retaliatory SPP. However, despite investigation of the cingulate subdivisions using different methodologies including electrical stimulation and high resolution magnetic resonance spectroscopy (Caruana et al., 2018; Dou et al., 2013), the nomenclature of the different cingulate subdivisions remains heterogeneous and thus precludes a clear understanding each subdivision's specific role. The pMCC was the only finding in the retaliatory SPP analysis. This might be due to the heterogeneity of the studies included which can lead to a lot of variability in regions activated across experiments. We only included 19 studies on retaliatory SPP in our meta‐analysis reporting results of different tasks (e.g., TAP, PSAP), contrasts, and different punishment modalities (e.g., money subtraction, noise blasts). More research is needed to reliably characterize the neural architecture of retaliatory SPP, for instance by mechanistically parsing out provocation from decision phases and by distinguishing among different punishment modalities and types of tasks. Our analyses showed activation of other areas during the feedback phase that did not emerge in the main analysis such as the aI/IFG, also active in altruistic SPP, as well as the superior temporal gyrus (STG) and the precentral gyrus (PreCG). However, no region emerged from the analysis on the decision phase, probably reflecting interindividual differences in the decision to punish. It is important to bear in mind the limited number of experiments included in these analyses, which thus limits the interpretation of the results. Nevertheless, it is likely that a more extended network than just the pMCC is consistently involved in retaliatory SPP. For instance, previous meta‐analytic findings on aggression (Wong et al., 2019) found convergent activation in the left postcentral gyrus. The postcentral gyrus is part of the somatosensory network (Kropf, Syan, Minuzzi, & Frey, 2019) and reflects action execution (Wong et al., 2019). However, in their meta‐analysis they only included 13 studies as opposed to the 19 studies included in the present work. The lack of convergent activation in this area in our meta‐analysis might indicate that this region is differently recruited during retaliatory SPP tasks, depending on heterogeneous decision‐making processes, which might be modulated by other brain areas as for example, the cerebellum as suggested by Klaus and Schutter (2021). The results from the subanalysis including only the TAP studies (see Table S4 and Figure S4) further suggests that tasks heterogeneity in retaliatory SPP may be responsible for convergent activation in the pMCC only. Indeed, the TAP studies did not show activation in the pMCC but in the bilateral STG and in the left precentral gyrus, which could reflect task‐specific patterns. The STG has been associated with looming threat (Blair et al., 2021), while the precentral gyrus is considered a motor mirror region (Gatti et al., 2017; Leslie, Johnson‐Frey, & Grafton, 2004).

### 4.1 Limitations

Some limitations should be considered in the interpretation of our results. First, the ALE algorithm is currently not accounting for variables differing between studies that might influence the results, such as scanning or data‐analysis parameters. The algorithm does, however, account for between‐studies variability by estimating the degree of spatial uncertainty in each experiment (Eickhoff et al., 2009). Second, we included both feedback phase and decision phase contrasts. This results in the inclusion of decision‐making processes in response to provocations that not always end up in a punishment and may thus to a certain degree potentially include decisions to avoid punishing the opponent. The number of studies and experiments available in the literature restricts the possibility to separately analyze with sufficient power the experiments, precluding reliable and robust separate computations of unfair > fair and reject > accept in altruistic SPP and feedback phase and decision phase in retaliatory punishment. Future studies should make a distinction between the two phases and the decision‐processes that lead to punishment and those that do not. Lastly, the categorization of the paradigms into altruistic and retaliatory scenarios may be artificial to a certain degree, as the differences in the decision process are very subtle and may in certain situations even overlap, for instance in scenarios in which the rejection of a gain (altruistic) goes along with a high punishment for the other (retaliation). Additionally, the decision‐making processes underlying retaliatory or altruistic motivations may include several cognitive processes beyond the presented situation, for example, evaluation of the situation and strategic thinking about long‐term outcomes. There are some differences between the two types of paradigms that might explain different brain activation beyond the altruistic and retaliatory motivations. For instance, retaliatory paradigms involve an interactive competition, in which the game outcome determines whether participants win money and administer the punishment or lose and receive the punishment. The UG, instead, is a strategic bargaining paradigm with a fixed role for the participant, that can only accept or reject the offer. The participant can never propose an offer and has a fixed cost of the punishment (gaining no money). Thus, for a more detailed understanding and replication of the differences suggested by this meta‐analysis, experimental designs that directly differentiate between differently motivated decisions and the parallel assessment of the reason the participant relied on, would be optimal.

## 5 CONCLUSION

Our findings show that SPP activates the anterior insula/IFG (detection and decoding of the norm violation), the MCC/dACC (integration of the salient information and selection of the appropriate response) and the dlPFC. The latter is likely primarily recruited in situations where a conflict needs to be resolved with suppression of self‐interest to implement the punishment (altruistic SPP). On the contrary, the pMCC is probably involved when a harming punishment comes at no direct cost for the participant (retaliatory SPP). With reduced conflict, the punishment may be channeled more directly. Our meta‐analysis integrates the previous findings on decision‐making in altruistic and retaliatory SPP. It also highlights that, despite of highly similar behavioral outcomes, different cognitive and emotional processes may underlie altruistic and retaliatory SPP. Our findings largely overlap with the norm violation model in the context of altruistic SPP and highlight the importance of clarifying the structural and functional subdivisions of the cingulate cortex, which seems to play a crucial role in differentiating the motivation supporting either altruistic or retaliatory SPP. These findings may drive future clinical studies aiming to understand which neural mechanisms are affected in pathological aggression and different SPP behaviors.

## CONFLICT OF INTERESTS

The authors declare no conflicts of interest.

## AUTHOR CONTRIBUTION

**Sara Boccadoro:** Designed the study, performed the database search, performed data analysis, interpretation, and wrote the manuscript. **Lisa Wagels:** Designed the study, revised the manuscript and provided critical feedbacks. **Andrei A. Puiu:** Designed the study, revised the manuscript and provided critical feedbacks. **Mikhail Votinov:** Revised the manuscript and provided critical feedbacks. **Carmen Weidler:** Revised the manuscript and provided critical feedbacks. **Tanja Veselinovic:** Revised the manuscript and provided critical feedbacks. **Zachary Demko:** Performed the database search. **Adrian Raine:** Revised the manuscript and provided critical feedbacks. **Irene Neuner:** Revised the manuscript and provided critical feedbacks. All authors contributed to and approved the final manuscript version.

## Supporting information


**Appendix S1**: Supporting Information

## Data Availability

The data that support the findings of this study are available upon request from the corresponding author.
