# Supplementary material for: A meta‐analysis on shared and distinct neural correlates of the decision‐making underlying altruistic and retaliatory punishment
Source: Hum Brain Mapp. 2021 Aug 20;42(17):5547–62. doi: 10.1002/hbm.25635 (PMC8559514; doi:10.1002/hbm.25635)
Supplement: Supplementary file 1 — Appendix S1: Supporting Information [file HBM-42-5547-s001.docx]

**Supplementary Table 1.**

*Results for the meta-analysis on Unfair > Fair contrast for altruistic SPP (22 experiments)*

| n | Brain regions | BA | MNI coordinates (mm) | | |  |  |
| --- | --- | --- | --- | --- | --- | --- | --- |
|  |  |  | x | y | z | ALE score | k |
| #1 | R dACC/aMCC | 32 | 8 | 26 | 34 | 0.0392 | 5144 |
|  | L SFG | 6 | -4 | 18 | 48 | 0.0388 |  |
|  | R dACC/aMCC | 32 | 8 | 30 | 24 | 0.0262 |  |
| #2 | R aI/IFG |  | 38 | 22 | -4 | 0.0424 | 4856 |
|  | R aI |  | 32 | 26 | 2 | 0.0407 |  |
| #3 | L aI |  | -30 | 22 | 2 | 0.0413 | 3896 |
|  | L IFG | 47 | -32 | 22 | -16 | 0.0195 |  |
|  | L IFG | 47 | -46 | 18 | -12 | 0.0153 |  |
| #4 | R MFG | 9 | 40 | 34 | 26 | 0.0251 | 1216 |
| #5 | L dACC/aMCC | 32 | -6 | 36 | 24 | 0.0218 | 1144 |
|  | L MFG | 9 | -6 | 32 | 32 | 0.0190 |  |

*Note*. n: cluster number; BA: Brodmann area; k: cluster size; R: right; L: left; aI: anterior insula; dACC: dorsal anterior cingulate cortex; aMCC: anterior midcingulate cortex; SFG: superior frontal gyrus; MFG: medial frontal gyrus; IFG: inferior frontal gyrus.


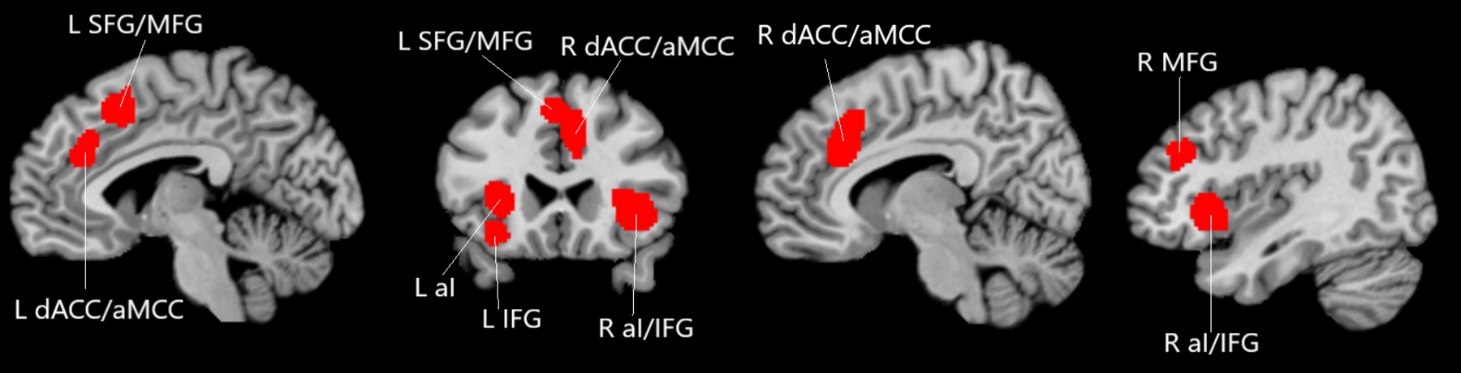


**Supplementary Figure 1.** Results for the meta-analysis on Unfair > Fair contrast for altruistic SPP (22 experiments). L: left; R: right; dACC: dorsal anterior cingulate cortex; aMCC: anterior midcingulate cortex; aI: anterior insula; SFG: superior frontal gyrus; MFG: medial frontal gyrus; IFG: inferior frontal gyrus.

**Supplementary Table 2.**

*Results for the meta-analysis on Reject > Accept contrast for altruistic SPP (9 experiments)*

| n | Brain regions | BA | MNI coordinates (mm) | | |  |  |
| --- | --- | --- | --- | --- | --- | --- | --- |
|  |  |  | x | y | z | ALE score | k |
| #1 | L aI | 13 | -32 | 20 | 4 | 0.0140 | 1616 |
|  | L putamen |  | -24 | 12 | 2 | 0.0129 |  |
|  | L IFG |  | -36 | 20 | 20 | 0.0120 |  |
|  | L aI/IFG | 13 | -36 | 16 | 14 | 0.0097 |  |
|  | L aI | 13 | -36 | 16 | -4 | 0.0092 |  |

*Note*. n: cluster number; BA: Brodmann area; k: cluster size; L: left; aI: anterior insula; IFG: inferior frontal gyrus.


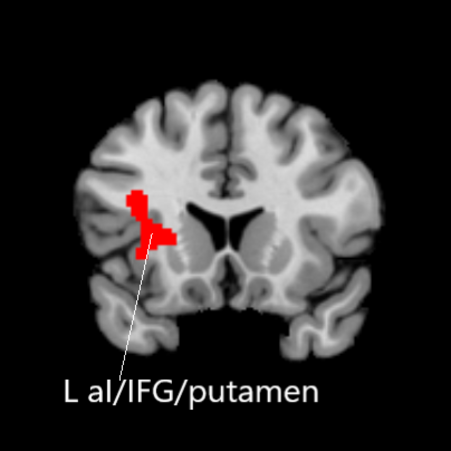


**Supplementary Figure 2.** Results for the meta-analysis on Reject > Accept contrast for altruistic SPP (9 experiments). L: left; aI: anterior insula; IFG: inferior frontal gyrus.

**Supplementary Table 3.**

*Results for the meta-analysis on the feedback phase for retaliatory SPP (9 experiments)*

| n | Brain regions | BA | MNI coordinates (mm) | | |  |  |
| --- | --- | --- | --- | --- | --- | --- | --- |
|  |  |  | x | y | z | ALE score | k |
| #1 | L PreCG | 44 | -48 | 2 | 4 | 0.0181 | 1824 |
|  | L aI |  | -38 | 8 | 0 | 0.0168 |  |
|  | L STG |  | -56 | 4 | -2 | 0.0158 |  |
|  | L aI/IFG | 13 | -34 | 16 | 8 | 0.0106 |  |
| #2 | R aI | 13 | 42 | 14 | 4 | 0.0158 | 792 |
|  | R aI | 13 | 46 | 6 | 4 | 0.0121 |  |
| #3 | R pMCC | 24 | 8 | 10 | 36 | 0.0130 | 664 |
|  | R pMCC | 24 | 8 | 10 | 44 | 0.0110 |  |
|  | R pMCC | 24 | 6 | 2 | 32 | 0.0100 |  |
| #4 | R Thalamus |  | 10 | -18 | -2 | 0.0155 | 584 |
| #5 | R SFG/MFG | 9 | 32 | 40 | 28 | 0.0139 | 544 |

*Note*. n: cluster number; BA: Brodmann area; k: cluster size; R: right; L: left; PreCG: precentral gyrus; aI: anterior insula; STG: superior temporal gyrus; IFG: inferior frontal gyrus; pMCC: posterior midcingulate gyrus; SFG: superior frontal gyrus; MFG: medial frontal gyrus.


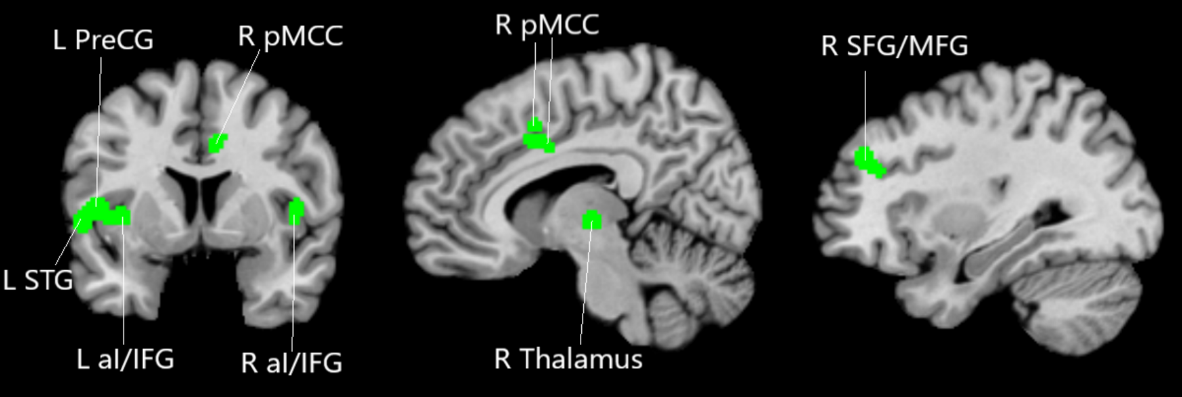


**Supplementary figure 3.** Results for the meta-analysis on the feedback phase for retaliatory SPP (9 experiments). L: left; R: right; pMCC: posterior midcingulate cortex; aI: anterior insula; SFG: superior frontal gyrus; MFG: medial frontal gyrus; IFG: inferior frontal gyrus.; STG: superior temporal gyrus; PreCG: precentral gyrus.

**Supplementary Table 4.**

*Results for the meta-analysis on retaliatory SPP with only TAP studies (20 experiments).*

| n | Brain regions | BA | MNI coordinates (mm) | | |  |  |
| --- | --- | --- | --- | --- | --- | --- | --- |
|  |  |  | x | y | z | ALE score | k |
| #1 | L PreCG | 44 | -48 | 2 | 4 | 0.0182 | 696 |
|  | L STG |  | -56 | 4 | -2 | 0.0159 |  |
| #2 | R STG | 22 | 56 | -8 | 2 | 0.0155 | 648 |
|  | R STG | 22 | 58 | -2 | -4 | 0.0149 |  |

*Note*. n: cluster number; BA: Brodmann area; k: cluster size; L: left; R: right; PreCG: precentral gyrus; STG: superior temporal gyrus.

**
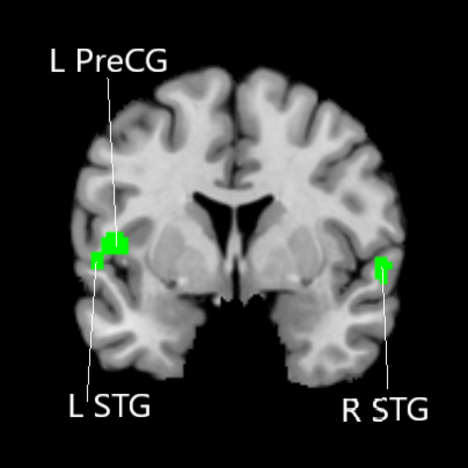
**

**Supplementary figure 4.** Results for the meta-analysis on retaliatory SPP with only TAP studies (20 experiments). L: left; R: right; STG: superior temporal gyrus; PreCG: precentral gyrus.
